# A *Chlamydia* effector recruits CEP170 to reprogram host microtubule organization

**DOI:** 10.1242/jcs.169318

**Published:** 2015-09-15

**Authors:** Maud Dumoux, Anais Menny, Delphine Delacour, Richard D. Hayward

**Affiliations:** 1 Institute of Structural and Molecular Biology, Birkbeck and University College London, Malet Street, London WC1E 7HX, UK; 2 Cell Adhesion and Mechanics Group, Institut Jacques Monod, CNRS UMR7592, Université Paris Diderot, 15 rue Helene Brion, Paris 75013, France

**Keywords:** Microtubules, Centrosome, *Chlamydia*, CEP170, Type III secretion

## Abstract

The obligate intracellular bacterial pathogen *Chlamydia trachomatis* deploys virulence effectors to subvert host cell functions enabling its replication within a specialized membrane-bound compartment termed an inclusion. The control of the host cytoskeleton is crucial for *Chlamydia* uptake, inclusion biogenesis and cell exit. Here, we demonstrate how a *Chlamydia* effector rearranges the microtubule (MT) network by initiating organization of the MTs at the inclusion surface. We identified an inclusion-localized effector that is sufficient to interfere with MT assembly, which we named inclusion protein acting on MTs (IPAM). We established that IPAM recruits and stimulates the centrosomal protein 170 kDa (CEP170) to hijack the MT organizing functions of the host cell. We show that CEP170 is essential for chlamydial control of host MT assembly, and is required for inclusion morphogenesis and bacterial infectivity. Together, we demonstrate how a pathogen effector reprograms the host MT network to support its intracellular development.

## Abstract (precis)

**Highlighted Article:** *Chlamydia trachomatis* is an obligate intracellular pathogen. Here, we describe how the *Chlamydia* virulence protein IPAM interacts with the host protein CEP170 to reprogram host MT organization.

## INTRODUCTION

*Chlamydiae* are obligate intracellular pathogens that remain the leading bacterial cause of sexually transmitted disease worldwide and blinding trachoma in developing nations. Increasing evidence also suggests that chlamydial infection contributes to tumorigenic processes in cervical cancer (Smith et al., 2004; Koskela et al., 2000).

Adherent chlamydial elementary bodies (EBs) employ a type III secretion system (T3SS) to deliver effectors that induce the reorganization of the actin cytoskeleton, promoting membrane deformation that triggers EB uptake into target cells. After internalization, individual EBs are encapsulated within vacuoles derived from the host plasma membrane. These vacuoles are rapidly diverted from the endocytic pathway and instead traffic to the perinuclear region, where they coalesce to form a larger specialized compartment termed the inclusion. In this environment, the bacteria differentiate into reticulate bodies and replicate actively (Abdelrahman and Belland, 2005).

A family of hydrophobic T3SS effectors called inclusion proteins (Incs) localize to the boundary membrane of the inclusion. Incs are key mediators of *Chlamydia*−host interactions together with other effectors that are delivered into the host cytosol and nucleus (Peters et al., 2007). Although most effector activities remain uncharacterized, inclusion biogenesis and bacterial growth are dependent upon the recruitment of Golgi-derived vesicles (Carabeo et al., 2003), multivesicular bodies (Beatty, 2006), lipid droplets (Cocchiaro et al., 2008) and the rough endoplasmic reticulum (Dumoux et al., 2012).

In addition to hijacking host organelles, *Chlamydia* also exploits the cytoskeleton to support its lifecycle. For instance, the T3SS effector Tarp is translocated during entry and promotes actin polymerization beneath the plasma membrane both directly and indirectly by stimulating the Arp2/3 complex (Jewett et al., 2006; Lane et al., 2008). An actin and intermediate filament cage is required for the integrity of mature inclusions (Kumar and Valdivia, 2008), and an acto-myosin-dependent process regulates inclusion extrusion from the infected cell (Hybiske and Stephens, 2007). Early *Chlamydia-*containing vacuoles utilize microtubules (MTs) to migrate from the cell periphery towards the MT-organizing center (MTOC) (Richards et al., 2013). Src family kinases regulate the tight association of the nascent and later mature *C. trachomatis* inclusion with the centrosome (Richards et al., 2013; Mital et al., 2010; Grieshaber et al., 2006), and disruption of this interaction forces the bacteria to enter a state of persistence (Romano et al., 2013; Leonhardt et al., 2007). Additionally, *C. trachomatis* induces supernumerary centrosomes by disrupting the centrosome duplication pathway (Johnson et al., 2009). MTs are also recruited in the vicinity of the chlamydial inclusion (Al-Younes et al., 2011), and MT-dependent transport processes sequester secretory traffic into the mature inclusion (Carabeo et al., 2003). However, how MT organization is initiated and controlled by *C. trachomatis* remains unknown. We hypothesized that a chlamydial Inc protein is important to initialize MT organization at the inclusion surface.

Here, we identified IPAM as an inclusion protein acting on MTs. We demonstrated that ectopic expression of the predicted cytosol-exposed C-terminal domain (CTD) of this Inc (IPAM-CTD) is sufficient to disturb the MT organizing activity of the cell. We used purified full-length IPAM in pull-down assays and identified the centrosomal protein 170 kDa (CEP170) as a host target by mass spectrometry. IPAM-CTD was also sufficient for CEP170 interaction. Next, we demonstrated that CEP170 is a key factor for chlamydial control of MT assembly, a role not ascribed to this protein in non-infected resting cells. CEP170 additionally influences inclusion morphogenesis, host cell shape and chlamydial infectivity. We show that endogenous IPAM and CEP170 act together to promote MT assembly from the inclusion. Together, our data demonstrate the ability of a virulence effector to manipulate the MT network to support intracellular bacterial development.

## RESULTS

### IPAM^−^ a *Chlamydia* inclusion protein acting on MTs

**Fig. 1.**
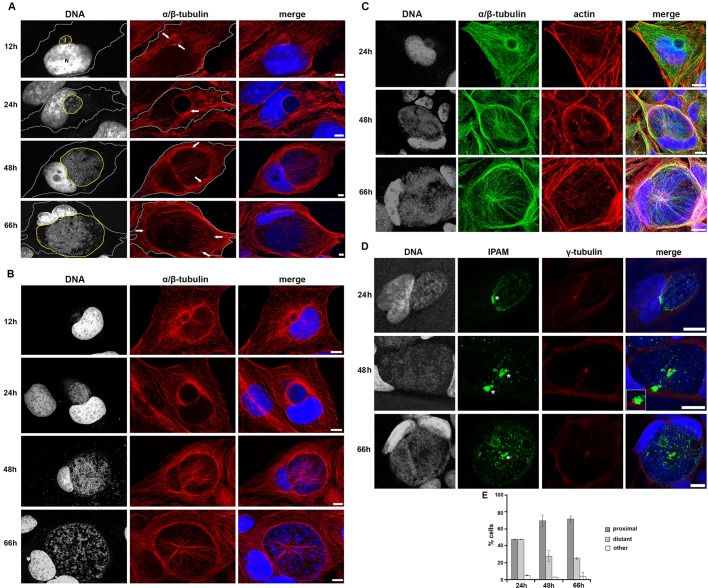
**Cytoskeletal organization and γ-tubulin positioning in cells infected with *C. trachomatis*.** (A,B) HeLa cells (A) or U2OS cells (B) were infected with *C. trachomatis* and incubated for 12, 24, 48 or 66 h, prior to fixation and staining for α/β-tubulin (red) and DNA (gray, blue in merged panel). Panels show maximum projection of four *z*-slices. In A, the cell periphery and the inclusion are outlined in grey and yellow, respectively. Arrows indicate the MT scaffold. Scale bars: 5 µm. (C) HeLa cells were infected with *C. trachomatis* and fixed after 24 h, 48 h or 66 h. Cells were stained for DNA (gray, blue in merged panel), α/β-tubulin (green) and actin (red). Panels show maximum projections obtained from the four *z*-sections shown. Scale bars: 10 µm. (D) HeLa cells were infected with *C. trachomatis* and fixed after 24 h, 48 h or 66 h. Cells were stained for DNA (gray, blue in merged panel), endogenous IPAM (green) and γ-tubulin (red). Patches of endogenous IPAM are visible at the inclusion membrane as described in supplementary material Fig. S2A. Asterisks indicate IPAM patches proximal to γ-tubulin foci. Inset shows the indicated region in a different *z*-section. Panels show maximum projection of four *z*-sections. Scale bars: 10 µm. (E) Quantification of the proximity of IPAM patches and the centrosome from confocal imaging (supplementary material Fig. S2A).

Using confocal microscopy, we initially confirmed the arrangement of the MT network during the maturation of the inclusion in detail in HeLa cells (Fig. 1A). At 12 h, MTs assemble at the inclusion periphery, and filaments partially cover this early structure. By 24 h, MTs have entirely surrounded the inclusion from where some filaments extend and contact the plasma membrane. Later (48−66 h), a dense MT scaffold encircles the inclusion. This scaffold is associated with a ‘nest’ of MTs that originate at the inclusion and extend towards the plasma membrane. The scaffold and nest MT superstructure was even more evident when human osteosarcoma cells (U2OS) were infected with *C. trachomatis* (Fig. 1B; Nans et al., 2014). Indeed, these cells have a larger cell volume and consequently a cytoskeleton that is easier to observe in comparison to HeLa cells. In particular, it was easier to discern at later time points (48−66 h) that MT actively accumulated around the inclusion periphery in the cell body rather than being compressed against the plasma membrane during inclusion expansion (compare Fig. 1A,B, 66 h). The initial MT scaffold present at 24 hours post infection (hpi), formed independently of the previously reported actin filament (F-actin) cage (Fig. 1C, 24 h), although inclusion-associated F-actin and MT structures coincided partially at later time points (Fig. 1C, 48 h and 66 h). Thus, host MTs are progressively organized at the inclusion surface, and assemble into an interlinked scaffold and nest superstructure.

The proximity of MTs and the inclusion suggested that a chlamydial effector located in the inclusion membrane might influence this process. As *Chlamydiae* encode Incs, we investigated whether any of these proteins shared sequence homology with MT-interacting proteins. Homology searches revealed that the predicted cytosol-exposed CTD, which encompasses residues 90−268 of Inc CT223, shares significant primary sequence similarity with human MT and centrosome-interacting proteins including pericentrin (supplementary material Fig. S1). In other Incs, similar interfaces enable host protein binding (Delevoye et al., 2008; Rzomp et al., 2006; Scidmore and Hackstadt, 2001). Provocatively, CT223 localizes to the inclusion membrane during infection (Bannantine et al., 2000), and the expression of CT223-CTD in cultured cells is correlated with cytokinesis defects (Alzhanov et al., 2009), a cellular process in which MT dynamics are key (D'Avino et al., 2005). We, hereafter, refer to CT223 as inclusion protein acting on MTs (IPAM).

Consequently, we initially investigated the localization of endogenous IPAM in cells infected with *C. trachomatis*, and any relationship to the host centrosome. We confirmed that IPAM is present at the inclusion membrane (Bannantine et al., 2000), throughout the infection time course (Fig. 1D). Strikingly, IPAM predominantly accumulated in defined patches at the inclusion periphery that frequently coincided with or were apposed to the centrosomes (Fig. 1D,E; supplementary material Fig. S2A). Centrosome supernumerary was also evident (Fig. 1D; Johnson et al., 2009).

**Fig. 2.**
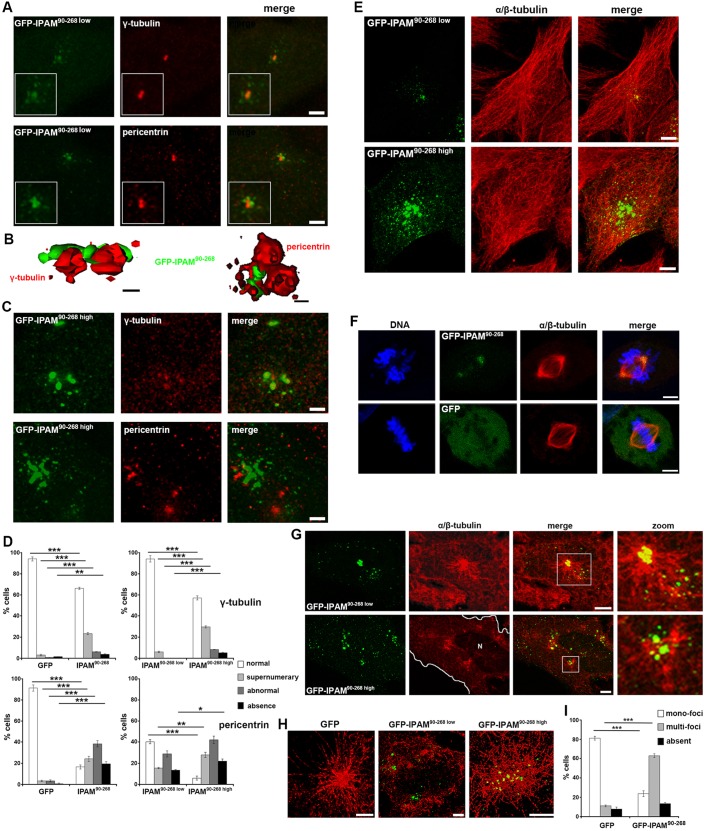
**Impact of GFP-IPAM^90-268^ on MT organization, centrosome integrity and MT assembly.** (A) HeLa cells expressing GFP-IPAM^90-268^ (green) were fixed and stained for γ-tubulin or pericentrin (both red). Fluorescence panels show maximum projection of three confocal *z*-sections spanning the centrosome. Scale bars: 5 µm. (B) 3D reconstructions from structural illumination microscopy (SIM). Scale bars: 2 µm. (C) Representative images of HeLa cells expressing high levels of GFP-IPAM^90-268^ (green) that had been fixed and stained for γ-tubulin or pericentrin (both red). Scale bars: 5 µm. (D) Following confocal imaging, the features indicated by γ-tubulin (top panels) or pericentrin (bottom panels) labeling were categorized (as shown in supplementary material Fig. S2B) using cells expressing GFP, GFP-IPAM^90-268 low^ or GFP-IPAM^90-268 high^. *P<0.05, **P<0.01, ***P<0.005. (E) HeLa cells expressing low or high levels of GFP-IPAM^90-268^ (green) were fixed and stained for α/β-tubulin (red). Panels show maximum projection of the entire *z*-stack. Scale bars: 5 µm. (F) HeLa cells were transfected with GFP-IPAM^90-268^ or GFP (green) and labeled for DNA (blue) and for α/β-tubulin (red). Panels show cells in division. Scale bars: 5 µm. (G) MTs were depolymerized in HeLa cells expressing GFP-IPAM^90-268^ (green) and allowed to regrow for 5 min prior to fixation and staining for α/β-tubulin (red). Panels show maximum projection of four confocal *z*-sections. Cell periphery is outlined in white, N indicates the nucleus. Scale bars: 5 µm. (H) MTs were depolymerized in HeLa cells expressing GFP, GFP-IPAM^90-268 low^ or GFP-IPAM^90-268 high^ (green). Panels show SIM reconstructions of MTs allowed to regrow for 5 min prior to fixation and staining for α/β-tubulin (red). The control GFP signal is absent because diffuse signals cannot be visualized by using SIM. Scale bars: 2 µm. (I) Following confocal imaging, MT architecture in individual cells expressing GFP or GFP-IPAM^90-268^ was categorized (as shown in supplementary material Fig. S2C) after MT depolymerization and 5 min of regrowth. ***P<0.005.

Given that endogenous IPAM patches are located at the inclusion periphery in proximity to the centrosome, we next investigated the effects of IPAM on the centrosome and MT organization in the absence of infection. We transiently expressed the CTD of IPAM (encompassing residues 90−268), termed IPAM^90-268^, as a GFP fusion (GFP-IPAM^90-268^) protein in cultured cells. GFP-IPAM^90-268^ localized with centrosomal proteins γ-tubulin and pericentrin (representative images in Fig. 2A), showing that the CTD of IPAM is sufficient to associate with the centrosome and, therefore, with the principal MTOC of the cell. Super-resolution structural illumination microscopy (SIM) revealed that GFP-IPAM^90-268^ is in close apposition to γ-tubulin and pericentrin, rather than colocalized, suggesting IPAM^90-268^ association with the pericentriolar matrix (PCM) and/or MT minus-ends (Fig. 2B). Moreover, GFP-IPAM^90-268^ expression induced centrosomal abnormalities including supernumerary of γ-tubulin and pericentrin puncta, and even the loss of defined centrosomes in some cells (Fig. 2C and supplementary material Fig. S2B). The most pronounced effect was on pericentrin distribution (Fig. 2D). Altogether, these data demonstrate that IPAM^90-268^ targets and destabilizes the host PCM.

As the main function of the MTOC is to assemble and organize MTs, we observed the MT network in cells transfected with IPAM^90-268^. At low expression levels, GFP-IPAM^90-268^ is located exclusively at the center of the MT array, which appeared structurally indistinguishable from that in control cells (Fig. 2E). However, increased GFP-IPAM^90-268^ expression provoked some MT disorder but did not affect F-actin architecture at steady-state (Fig. 2E and supplementary material Fig. S3A). In dividing cells, GFP-IPAM^90-268^ localized to the spindle pole and apparently partitioned preferentially to one centriole (Fig. 2F). This mobility confirmed the strong affinity of GFP-IPAM^90-268^ for the centrosome, which is retained even during physiological remodeling of the MT, and suggests that ectopically expressed IPAM^90-268^ is unlikely to be forming inactive cytosolic aggregates.

Given the effect of IPAM^90-268^ on the PCM and the robust relationship between the pericentriolar material and MT organization (Woodruff et al., 2014), we analyzed the impact of IPAM expression on MT assembly following MT depolymerization and regrowth. GFP-IPAM^90-268^ localization remained unchanged after MT disassembly (supplementary material Fig. S3B), indicating MT-independent association of IPAM^90-268^ with the centrosome. Moreover, the presence of GFP-IPAM^90-268^ did not interfere with MT depolymerisation upon induced disassembly (supplementary material Fig. S3C). After 5 min regrowth, GFP-IPAM^90-268^ remained enriched at the centrosome, and additional GFP-IPAM^90-268^ foci were observed along newly polymerized MTs (Fig. 2G). Higher levels of expressed GFP-IPAM^90-268^ generated multiple MT nucleation foci within individual cells (Fig. 2G). Nucleated MTs were non-homogenous in length and appeared to form a randomly oriented network. When examined at higher resolution by SIM, it was evident that even low-level GFP-IPAM^90-268^ expression was sufficient to alter nucleation foci, and generated shorter and interconnected emergent MT networks in comparison to those in GFP-control cells (Fig. 2H). Multi-foci MT networks were apparent in 62.9±1.2% of GFP-IPAM^90-268^ transfectants vs 11.1±1.6% of GFP-control cells (Fig. 2I). Thus, IPAM-CTD perturbs MT organization and assembly.

Therefore, we demonstrate that IPAM-CTD, which shares homology with eukaryotic centrosomal and MT-related proteins, targets the main cellular MTOC and destabilizes the PCM. Consequently, the MT organizing activity of the MTOC is disturbed, even when IPAM-CTD is expressed at low levels. Furthermore, when IPAM^90-268^ is overexpressed, ectopic MT nucleation foci are formed. Together, these data show that IPAM associates with the PCM at the centrosome and is sufficient to hijack the MT organizing function of the host cell.

### *Chlamydia* IPAM interacts with host CEP170 to perturb MT assembly

**Fig. 3.**
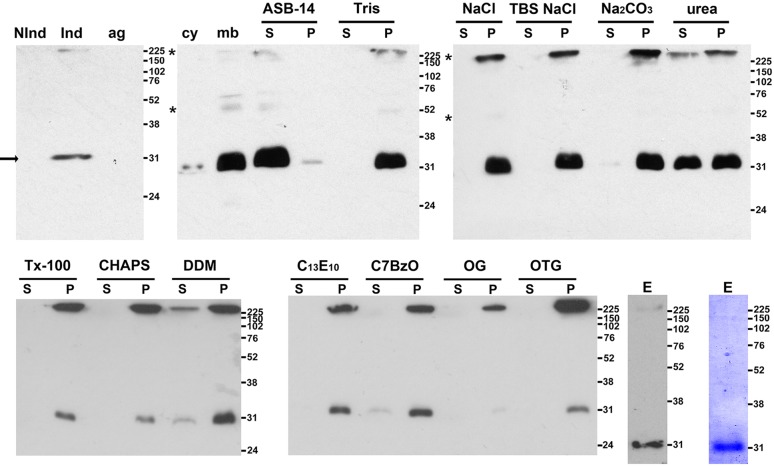
**Affinity purification of native IPAM from *E. coli*.** Immunoblots obtained using anti-His_6_ antibody of SDS-PAGE-separated fractions from *E. coli* transformants expressing IPAM. Cell lysates before (Nind) and after induction with IPTG (Ind) are shown together with a pellet obtained following low-speed centrifugation to remove aggregated proteins and unlysed cells (ag), bacterial cytosol (cy) and membrane (mb) fractions. Membranes were incubated with buffers [20 mM Tris-Cl pH 7.4 (Tris); 20 mM Tris-Cl pH 7.4, 140 mM NaCl (NaCl); 20 mM Tris-Cl pH 7.4, 1 M NaCl (Tris NaCl); 100 mM Na_2_CO_3_ pH 11 (Na_2_CO_3_)]; 8 M urea (urea) or detergents in TBS [1% amidosulfobetaine-14 (ASB-14), Triton X-100 (Tx-100); CHAPS, dodecylmaltoside (DDM); polyoxyethylene 10 tridecylether (C_13_E_10_); C7BzO; octylglucoside (OG); and octylthioglucoside (OTG)] prior to centrifugation to separate solubilized proteins in the supernatant (S) from the membrane pellet (P). IPAM is present at the expected molecular mass (32 kDa, arrow) and multimeric IPAM is also evident (asterisk). Immunoblot and Coomassie-Blue-stained immunoblot of eluate (E) after affinity purification in 1% ASB-14; kDa values for molecular mass markers are shown on the right of all panels.

To identify eukaryotic interacting partners using a pull-down approach, we expressed IPAM in *Escherichia coli*. Full-length hydrophobic IPAM partitioned predominantly to the bacterial membrane fraction (Fig. 3), from where it could only be solubilized with detergent, indicating insertion of IPAM into the *E. coli* membrane. Further purification was carried out under native conditions (Fig. 3; see Materials and Methods). Exploiting the affinity tag, we performed a pull-down assay using purified IPAM and human cell extract (see Materials and Methods), and identified 12 candidate-interacting proteins by mass spectrometry. Of these, six were MT and centrosome related, the most significant being centrosomal protein 170 (CEP170; Mascot score 65.56, two peptides read ten times each). CEP170 is a component of the mother centriole that interacts with kinesin-like motors during cell division (Maliga et al., 2013; Welburn and Cheeseman, 2012; Guarguaglini et al., 2005). Furthermore, CEP170 overexpression disturbs centrosomal positioning and the MT network (Guarguaglini et al., 2005). Given the similar effects of IPAM-CTD expression on the centrosome and MTs (Fig. 2), we focused on examining potential interplay between IPAM and CEP170 during *C. trachomatis* inclusion biogenesis.

**Fig. 4.**
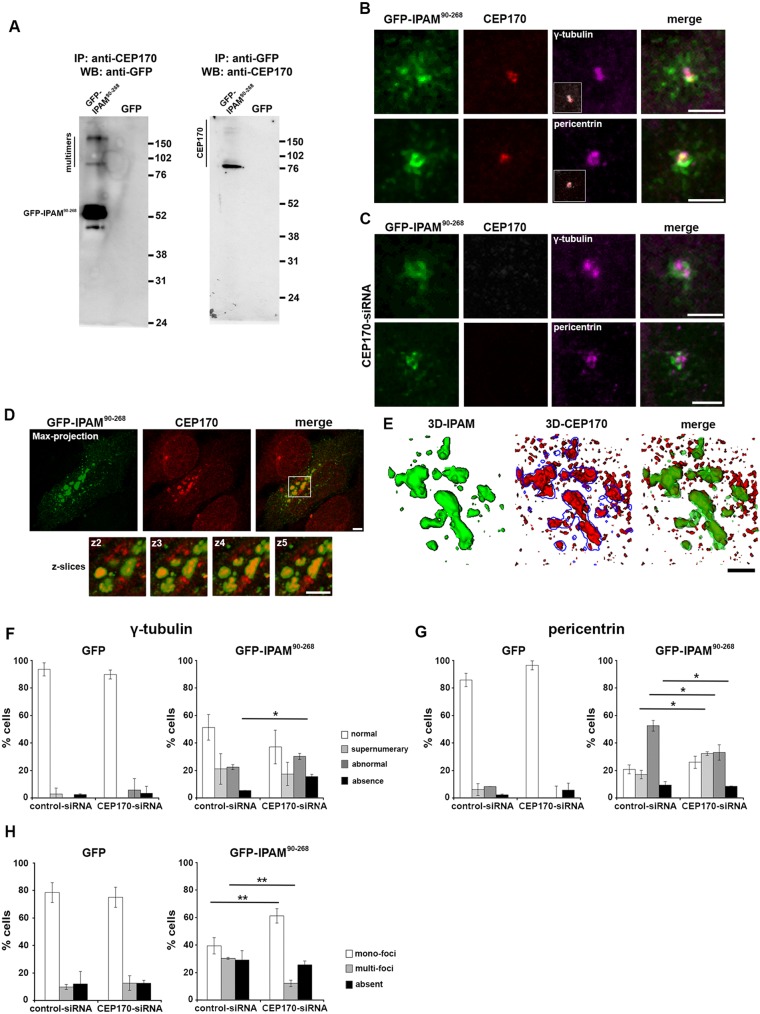
**Functional interaction between GFP-IPAM^90-268^ and CEP170.** (A) Western blots (WB) immunostained with anti-CEP170 or anti-GFP antibodies of SDS-PAGE-separated proteins immunoprecipitated (IP) with anti-CEP170 or anti-GFP antibodies. IPAM multimers are evident (see Fig. 3). Positions of molecular mass markers are indicated in kDa (right). (B) HeLa cells expressing low levels of GFP-IPAM^90-268^ (green) were fixed and co-stained for γ-tubulin or pericentrin (magenta) and CEP170 (red). Insets show CEP170 (red) and γ-tubulin or pericentrin (gray) labeling only. Scale bars: 2 µm. (C) HeLa cells treated with CEP170-siRNA and expressing GFP-IPAM^90-268^ (green) were fixed and co-stained for γ-tubulin or pericentrin (magenta) and CEP170 (red). Scale bars: 2 µm. (D) HeLa cells expressing high levels of GFP-IPAM^90-268^ (green) were fixed and stained for CEP170 (red). Panels show maximum projections of four confocal *z*-sections (left). Scale bar: 5 µm. Smaller panels below show indicated region at higher magnification (right) broken down into individual *z*-sections (2−5). Scale bar: 2 µm. (E) 3D reconstructions from structural illumination microscopy, demonstrating that GFP-IPAM^90-268^ colocalizes with CEP170. Scale bar: 2 µm. (F,G) γ-tubulin (F) or pericentrin (G) labeling was categorized (as described in supplementary material Fig. S2B) in HeLa cells treated with CEP170-siRNA or control-siRNA expressing GFP or GFP-IPAM^90-268^ as indicated. *P<0.05. (H) Quantification of the MT network (as described in supplementary material Fig. S2C) in HeLa cells treated with CEP170-siRNA or control-siRNA expressing GFP or GFP-IPAM^90-268^ as indicated. **P<0.01.

GFP-IPAM^90-268^ was isolated following immunoprecipitation of CEP170 from transfected cells (Fig. 4A). Reciprocally, endogenous CEP170 was purified when GFP-IPAM^90-268^ was immunoprecipitated from transfectants (Fig. 4A). Thus, the IPAM-CTD is sufficient for CEP170 interaction in cells. Immunostaining of endogenous CEP170 in GFP-IPAM^90-268^ transfectants demonstrated colocalization of both proteins at the centrosome (Fig. 4B), consistent with our *in vitro* association data.

Next, we investigated whether CEP170 is required for centrosomal localization of IPAM^90-268^. CEP170 knockdown was confirmed by immunofluorescence and immunoblotting (supplementary material Fig. S3D,E). Silencing of CEP170, did not release GFP-IPAM^90-268^ from the centrosome, and γ-tubulin and pericentrin localization remained similarly undisturbed (Fig. 4C), in agreement with previous observations (Guarguaglini et al., 2005). Nevertheless, higher levels of GFP-IPAM^90-268^ expression promoted CEP170 redistribution into additional extra-centrosomal foci, where both proteins colocalized (Fig. 4D and E). Although CEP170 is, therefore, not necessary for the association of GFP-IPAM^90-268^ with the centrosome, GFP-IPAM^90-268^ expression is sufficient to influence CEP170 localization in cells.

Given this dynamic interplay between GFP-IPAM^90-268^ and CEP170, we determined whether CEP170 is required for GFP-IPAM^90-268^-mediated disruption of the centrosome. We examined γ-tubulin and pericentrin in cells expressing control siRNA or CEP170 siRNA in the presence or absence of GFP-IPAM^90-268^, and classified the resultant centrosomal structures (supplementary material Fig. S2B). The nature of the defects particularly in the PCM reported by pericentrin was subtly, yet significantly, altered in cells transfected with CEP170 siRNA (Fig. 4F,G), demonstrating a partial role for CEP170 in IPAM-mediated PCM interference. Similarly, we investigated whether CEP170 is necessary for IPAM^90-268^ to disturb MT organization (Fig. 4H). CEP170 knockdown did not influence the MT organization in GFP-control cells but partially rescues GFP-IPAM^90-268^-induced defects in MT assembly, restoring MT regrowth (Fig. 4H). Additionally, the normal MT organization during regrowth in CEP170 siRNA cells (i.e. in the absence of GFP-IPAM^90-268^) strongly suggests that CEP170 does not play a housekeeping role in MTOC function per se. Consequently, we conclude that the interaction between IPAM^90-268^ and CEP170 is functional.

Taken together, we show that IPAM interacts with the host protein CEP170. Whereas IPAM^90-268^ localization to the centrosome is CEP170-independent, the effect of IPAM^90-268^ on PCM and the MT organization is mainly CEP170-dependent.

### CEP170 influences MT organization through IPAM in infected cells

**Fig. 5.**
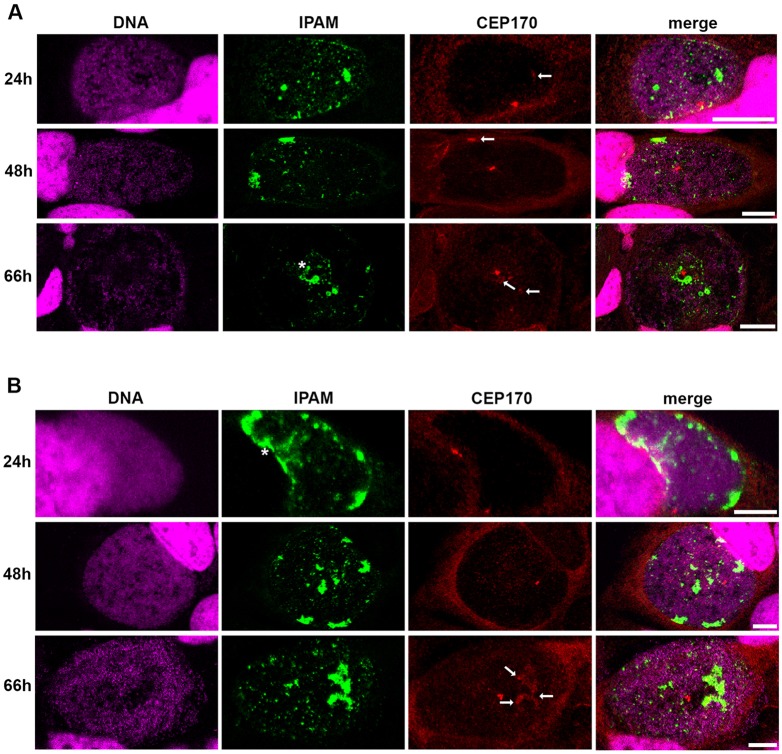
**IPAM and CEP170 location in *C. trachomatis*-infected cells.** (A,B) HeLa cells (A) or U2OS cells (B) were infected with *C. trachomatis* for 24 h, 48 h or 66 h and labeled for DNA (magenta), endogenous IPAM (green) and CEP170 (red). Asterisk indicates IPAM patches (as described in supplementary material Fig. S2A) in proximity of CEP170 dots. Arrows indicate diffuse CEP170 signal associated to IPAM. Scale bars: 10 µm.

As IPAM is sufficient for CEP170 interaction, we next examined the relative location of endogenous IPAM and CEP170 in cultured HeLa and U2OS cells infected with *C. trachomatis.* CEP170 was present in patches at the inclusion membrane from 24 hpi and multiple patches were evident at 66 hpi (Fig. 5). Consistent with the *in vitro* interaction and the effects of IPAM^90-268^ in cultured cells, colocalization of IPAM and CEP170 was observed at the inclusion, although – as might be expected during infection – both IPAM and CEP170 were additionally present in isolation (Fig. 5).

**Fig. 6.**
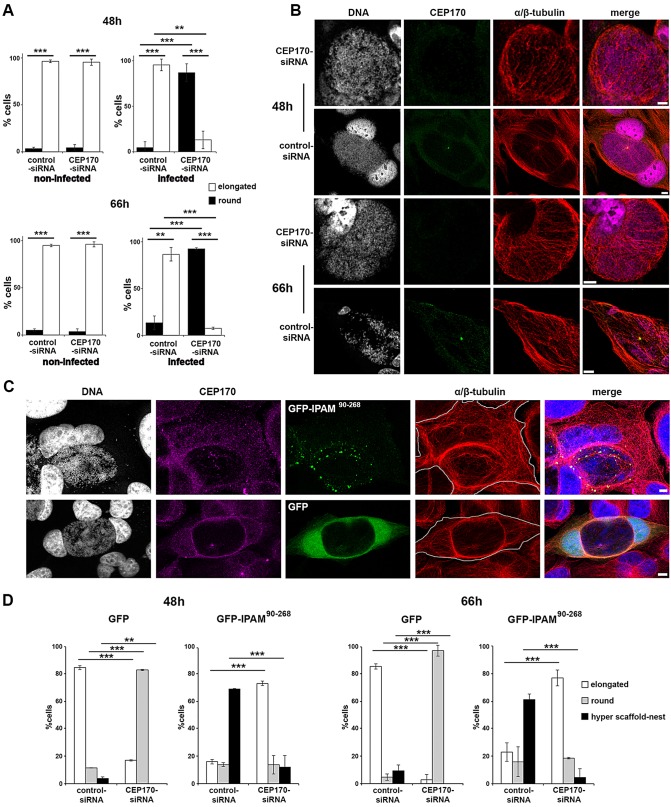
**CEP170-IPAM interaction is essential to maintain cell shape and MT organization in *Chlamydia-*infected cells.** (A) HeLa cells were transfected with control-siRNA or CEP170-siRNA, and infected with *C. trachomatis* where appropriate. Cell shape was assessed and categorized after 48 h and 66 h. Cells in mitosis identified by DNA staining were excluded. **P<0.01, ***P<0.005. (B) HeLa cells transfected with CEP170-siRNA or control-siRNA were infected with *C. trachomatis* and fixed after 48 h or 66 h. Cells were stained for DNA (grey), CEP170 (green) and α/β-tubulin (red). Panels show maximum projections of four *z*-sections. Scale bars: 10 µm. (C) HeLa cells were transfected with CEP170-siRNA or control-siRNA for 24 h, prior to infection with *C. trachomatis* and transfection with plasmid encoding GFP or GFP-IPAM^90-268^ (green). Cells were fixed 48 or 66 h later and labeled for DNA (gray, blue in merged panel) CEP170 (purple) and α/β-tubulin (red). Panels represent maximum projections of four *z*-sections in cells 48 h after infection, showing a representative ‘hyper scaffold-nest’. The cell periphery is outlined in gray. Scale bars: 5 µm. (D) Following confocal imaging, cells shape was categorized (as described in supplementary material Fig. S2E) for *C. trachomatis*-infected cells. ***P<0.005, **P<0.01.

Since CEP170 influences MT organization only in the presence of IPAM, we investigated the effect that silencing the expression of CEP170 has on MT organization during inclusion biogenesis. CEP170 knockdown remained stable for the duration of the infection cycle (supplementary material Fig. S3D,E), and did not significantly influence the early phase (<24 h) of bacterial development in cultured cells (supplementary material Fig. S3F). Although Nigg and co-workers have suggested that CEP170 knockdown in HeLa cells induces a change in cell shape from a cobblestone-like to an elongated phenotype (Guarguaglini et al., 2005), under our growth conditions, non-infected HeLa cells remained elongated independently of CEP170 knockdown (Fig. 6A). However, after infection with *C. trachomatis*, simultaneous CEP170 knockdown resulted in rounding of the infected cells, whereas infected control siRNA cells remained elongated at both 48 h and 66 h (Fig. 6A, supplementary material Figs S2E and S4A). This indicated a substantial CEP170-dependent change in host cytoskeletal organization in infected cells. To investigate this, we visualized the MT network in these two cell populations. In the infected control cells, the MT network displayed a radial organization where MTs emerge from the CEP170-positive MTOC to form the characteristic MT scaffold and nest. In equivalently infected CEP170 knockdown cells, there were profound alterations; a partial MT scaffold was formed by an array of parallel MTs without a distinct point of emergence; also, the MT nest was absent, driving the distinct rounded cell phenotype (Fig. 6B and supplementary material Fig. S4A). Nevertheless, the extent of colocalization between F-actin and the MT network surrounding the aberrant-shaped inclusion was not significantly affected in infected CEP170 siRNA cells (supplementary material Fig. S3G). Intriguingly in non-infected cells, the MT and F-actin networks in CEP170-knockdown cells were indistinguishable from controls at steady-state (supplementary material Fig. S3H), correlating with the absence of a change in cell shape in these two populations (Fig. 6A). This strongly suggests that the CEP170-dependent pathway stimulated by *C. trachomatis* to reorganize MTs is silent in resting uninfected cells or unconventional in infected cells.

To probe further how CEP170 functions relate to IPAM, we examined the consequences of GFP-IPAM^90-268^ overexpression in infected cells in the presence and absence of CEP170. When expressed in infected cells, GFP-IPAM^90-268^ puncta are enriched at the inclusion, with a minor population present at the cell periphery (Fig. 6C). Moreover, we observed a more prominent MT scaffold associates with a hyper-developed nest, modifying the cell shape in a distinctive manner in cell expressing endogenous levels of CEP170 (Fig. 6C and D). This exaggerated phenotype due to the presence of high levels of GFP-IPAM^90-268^ in infected cells is indicative of an IPAM-induced overstimulation rather than a dominant negative effect. In CEP170 siRNA cells, GFP-IPAM^90-268^ overexpression partially reverts the defect in the formation of the MT nest (Fig. 6D), suggesting that GFP-IPAM^90-268^ is able to compensate for loss of CEP170 via a secondary or redundant pathway.

These data reinforce our view that CEP170 function is extended in infected cells, and that this augmentation depends on IPAM^90-268^. Also, whereas IPAM^90-268^ preferentially acts with CEP170 to organize MTs, like with other bacterial effectors, IPAM can also stimulate redundant pathways – possibly through additional interacting partners – to reorganize MTs in the absence of CEP170.

### CEP170 is essential for MT assembly, inclusion morphology and *Chlamydia* replication

**Fig. 7.**
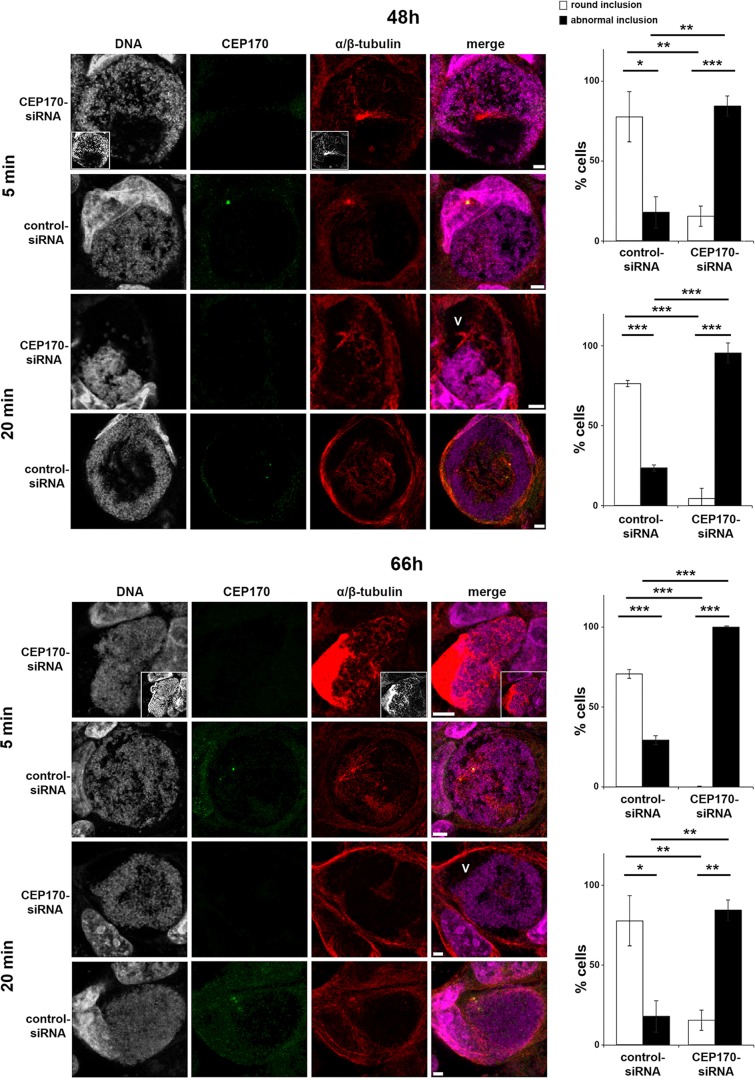
**CEP170 knockdown impairs MT assembly in cells infected with *C. trachomatis.*** HeLa cells were transfected with CEP170-siRNA or control-siRNA and infected with *C. trachomatis.* After 48 h (top panels) or 66 h (lower panels), MTs were depolymerized then allowed to regrow for 5 or 20 min prior to fixation. Cells were stained for DNA (gray, magenta in merged panels), CEP170 (green) and α/β-tubulin (red). Panels show maximum projections of four *z*-sections. Void (V) is marked within an aberrant inclusion. Scale bars: 10 µm. Histograms on the right show assessment of inclusion morphology (as described in supplementary material Fig. S2F) under the conditions indicated. ***P<0.005, **P<0.01, *P<0.05.

Given that CEP170 is a key determinant of MT organization during infection, we next examined MT regrowth to assess the dynamics of this mechanism (Fig. 7). CEP170 knockdown does not significantly influence MT regrowth in uninfected cells (supplementary material Figs S3I and S2D). In infected cells, newly polymerized MTs emerged from single or multiple MTOCs and encased the inclusion after 5 min. After 20 min, MT accumulation around the inclusion was less pronounced than at steady-state but the nest did reform (Fig. 7). After CEP170 knockdown, MTs emerged from an enlarged structure following 5 min regrowth at 48 h, and at 66 h tubulin dramatically accumulated at one pole of the inclusion under equivalent conditions (Fig. 7) demonstrating the crucial role for CEP170 in MT assembly. After 20 min regrowth, MTs are present as in control cells, but inclusions lost their typical spherical morphology and appear constricted (Fig. 7). Indeed, inclusion architecture was severely disrupted because voids – characterized by the absence of bacteria and MTs in the presence of an intact inclusion membrane – were evident (Fig. 7 and supplementary material Fig. S4B). This is interesting because it has been predicted, using experiment-based mathematical models, that contact between the bacteria and the inclusion membrane controls chlamydial development (Wilson et al., 2006; Hoare et al., 2008). Consequently, we investigated whether CEP170 is required for chlamydial infectivity. Although siRNA-mediated knockdown inherently generates a mixed population of cells that expresses varying amounts of CEP170 protein, CEP170 knockdown significantly (*P*<0.05) attenuated the infectivity of *C. trachomatis* progeny (12.3×10^4^±2.7×10^4^ in control siRNA vs 6.3×10^4^±2.3×10^4^ in CEP170 siRNA cells). These data demonstrate a key role for CEP170 in MT assembly at the inclusion periphery allowing the maintenance of inclusion shape and the success of the chlamydial infectious cycle.

### Interaction between endogenous IPAM and CEP170 plays a role in MT assembly within cells infected with *C. trachomatis*

**Fig. 8.**
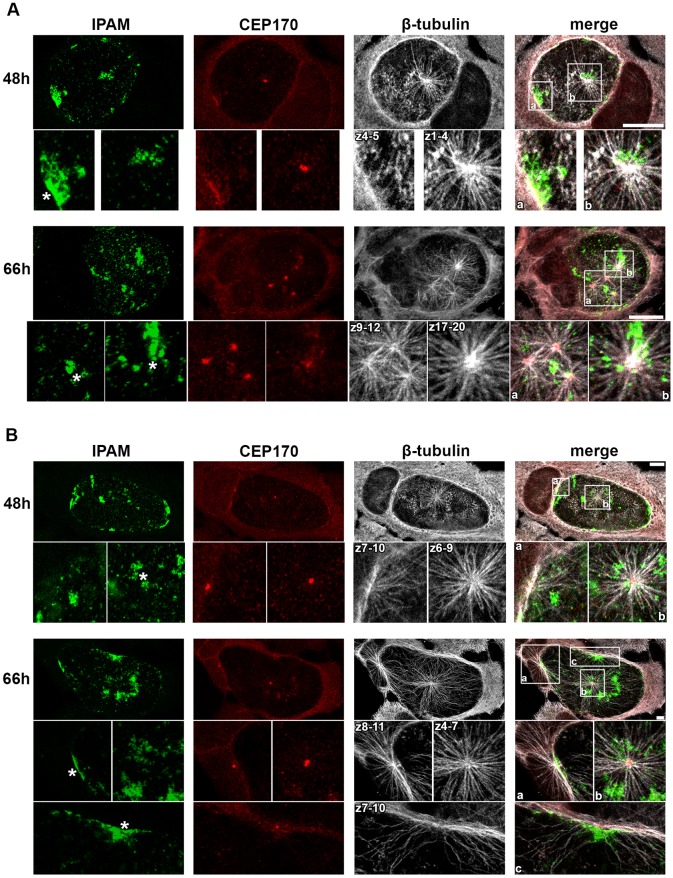
**Endogenous IPAM localizes to sites of MT assembly in *C. trachomatis*-infected cells.** (A,B) HeLa cells (A) or U2OS cells (B) were infected with *C. trachomatis* for 48 h or 66 h prior to MT regrowth for 5 min. Cells were fixed and labeled for endogenous IPAM (green), CEP170 (red) and β-tubulin (gray). Upper panels show maximum projections through the entire cell volume. Lower panels, show the maximum projection of the indicated *z*-section from the marked region of interest. Asterisks indicate the IPAM patches where MTs regrow. Scale bars: 10 µm.

Using transfected cells and knockdown approaches, we demonstrate that the IPAM-CTD perturbs MT assembly through its interaction with CEP170, which is essential for MT organization in infected cells. Next, we investigated the nature of the MT nucleation centers in infected cells, examining endogenous IPAM and CEP170. To facilitate the observation of the point of emergence of MTs, we performed an MT regrowth assay for 5 min. At 48 hpi, 75% of infected cells showed multiple points of MT nucleation on the inclusion, which were ubiquitously observed by 66 hpi in both HeLa and U2OS cells (Fig. 8A and B). Interestingly, these MTOCs are distributed across the 3D volume of the inclusions either individually or as groups (Fig. 8A,B, insets showing different *z* positions).

In both cell lines, ∼95% of inclusion-associated MT nucleation foci contained CEP170, either at defined points or within a more diffused pattern. Occasionally, patches of colocalized IPAM and CEP170 were observed without emerging MTs, although CEP170-positive MT nucleation foci can localize with IPAM patches in HeLa and U2OS cells (50% and 60%, respectively) (Fig. 8A,B), indicating the presence of the IPAM–CEP170 complex in at least 50% of inclusion-associated MT nucleation centers captured by using this method within infected cells. These data reveal that endogenous IPAM and CEP170 cooperate at the surface of the inclusion to orchestrate reorganization of host MTs.

## DISCUSSION

We have demonstrated here that *Chlamydia trachomatis* integrates the transmembrane effector IPAM into the inclusion membrane to locally hijack the host MT-organizing activity by recruiting CEP170. We propose that this event leads to the formation of a MT ‘scaffold and nest’ superstructure that is necessary to maintain cell and inclusion morphology and is required for bacterial infectivity. Moreover, we show that CEP170 exhibits additional roles in MT organization when compared to resting cells.

IPAM shares partial sequence sequence similarity with MT and centrosomal proteins. We confirmed that the *C. trachomatis* inclusion traffics to and associates with the MTOC (Richards et al., 2013; Grieshaber et al., 2006), and demonstrated the proximity of endogenous IPAM patches and the centrosome. When the IPAM-CTD is expressed in cells, it localizes to the centrosome, the principal MTOC. IPAM^90-268^ induces centrosomal abnormalities and has a pronounced effect on the organization of the PCM. This is intriguing considering that centrosomal dysfunction is correlated to aneuploidy and tumor initiation (Nigg, 2002), and that *C. trachomatis* infection is associated with cervical cancer (Smith et al., 2004; Koskela et al., 2000).

Consequently, the MTOC is able to generate MTs but their organization is disrupted. When IPAM^90-268^ is overexpressed, multiple MT nucleation centers become evident. These data show that IPAM is sufficient to reprogram the MT organizing activity of the host cell. Intriguingly, in cells infected with *C. trachomatis*, multiple MTOC were observed and we demonstrate that 50−60% of the CEP170-positive MT nucleation foci are associated with IPAM.

Although IPAM^90-268^ localization to the centrosome is CEP170 independent, IPAM-directed PCM disruption and MT reorganization is largely dependent on CEP170. This demonstrates functional interaction between IPAM^90-268^ and CEP170, and reveals CEP170 activities not previously observed in resting uninfected cells. These extended functions were supported by CEP170 knockdown. In our hands, uninfected cells that lack CEP170 exhibited no detectable abnormalities in cell shape, MT organization or MT assembly. By contrast, knockdown of CEP170 in cells infected with *C. trachomatis* resulted in a reduced MT scaffold and the absence of the MT nest, leading to a characteristic round cell shape. This was accompanied by defects in MT organization during regrowth and changes in inclusion morphology. Thus, CEP170 functions are exacerbated during *C. trachomatis* infection. Indeed, these additional functions highlighted by studying infection could indicate dormant or incompletely understood roles for CEP170 in uninfected non-dividing cells. Intriguingly, overexpression of IPAM^90-268^ in infected cells can maintain MT architecture in the absence of CEP170, showing that IPAM can functionally compensate using CEP170-independent secondary or redundant pathways, possibly by interacting with additional eukaryotic binding partners.

Our data, therefore, show that IPAM reorganizes MTs indirectly by interacting with CEP170 and possibly other eukaryotic partners. This is distinct from other bacterial effectors, including *Chlamydia trachomatis* CopN, which acts directly on tubulin subunits to inhibit polymerization (Archuleta et al., 2011). To our knowledge, this is the first example of such significant structural changes in organization of the MT network by an intracellular bacterial pathogen. *Salmonella*, *Brucella* and *Legionella* all exploit the MT network to commandeer vesicular transport but the associated MT architecture is not profoundly rearranged (Radhakrishman and Splitter, 2012). Only a toxin secreted by the extracellular bacterium *Clostridium* generates localized MT reorganization by inducing MT-derived protrusions (Schwan et al., 2009).

Recently, a population of specialized post-translationally modified MTs was observed surrounding the inclusion and is required for Rho-dependent tethering of Golgi mini-stacks. It was proposed that *C. trachomatis* must initially reorganize MTs that are subsequently detyrosinated 2–3 h later (Al-Zeer et al., 2014). When the inclusion expands over time, these specialized MTs must be continuously readjusted through a cycle of MT assembly and modification, as also suggested for the actin cage (Kumar and Valdivia, 2008). Our data mechanistically complement and extend this study, as we identify IPAM as an effector that initiates MT organization at the inclusion surface by recruiting CEP170. Subsequently, these MTs could be modified by unknown host or additional chlamydial factors, allowing Rho-dependent organelle positioning.

CEP170 binds MTs (Guarguaglini et al., 2005), but whether IPAM also engages MT directly or requires CEP170 remains unknown. Moreover, because Incs have the propensity to engage each other in heterotypic Inc–Inc complexes (Gauliard et al., 2015), additional Incs might interact with IPAM at the inclusion membrane and could regulate its activity. For instance, recent evidence suggests that the Inc protein CT850 binds the MT-associated motor protein dynein (Mital et al., 2015). Moreover, it is known that CEP170 is phosphorylated through Polo-like kinase 1 (Guarguaglini et al., 2005); but how the phosphorylation of CEP170 is involved in the formation or activities of the IPAM–CEP170 complex or influences chlamydial infection remains unknown. Further *in vitro* reconstitution experiments are now essential to understand the molecular mechanisms of IPAM- and CEP170-mediated MT and centrosomal subversion in detail, and to delineate the domains of each partner that is involved in their functional interactions.

## MATERIALS AND METHODS

### Sequence homology searches

The primary amino acid sequences of the 55 predicted inclusion proteins from *C. trachomatis* serovar L2 (Lutter et al., 2012) were compared to the *Homo sapiens* (taxid 9606) database using Blast (blast.ncbi.nlm.nih.gov/Blast.cgi). Results were manually filtered for known MT and/or centrosomal proteins, and ranked according to the number of identical and/or similar amino acid residues.

### Cell culture, transfection, knockdown and infection

HeLa or U2OS cells were routinely cultured in DMEM containing 10% (v/v) fetal calf serum and antibiotics [penicillin (100 U ml^−1^)/streptomycin (100 µg ml^−1^) or gentamicin (25 µg ml^−1^), Invitrogen]. Cells were transfected with pEGFP-C2 or pEGFP-IPAM^90-268^ (300 ng ml^−1^), using Turbofect according to the manufacturer's instructions (Fermentas). Cells were transfected with 10 nM of siRNA designed against CEP170 sequence or against non-specific sequence, using Hiperfect according to the manufacturer's instructions (Qiagen). Three siRNAs (5′-AAGCATGGAGATTTCTTCTAT-3′, 5′-AAAGTGTTTCTGGAACTTTAA-3′, 5′-TTGGATATGATACAAATCTTT-3′) directed against CEP170 were independently validated by using western blot and immunofluorescence. Cells were infected with *C. trachomatis* serovar L2 as previously described (Dumoux et al., 2012). When these approaches were used in combination, cells (density 4×10^4^ cm^−2^, plated 24 h previously) were transfected with plasmids immediately prior to infection (*t*=0 h), or with siRNA 24 h prior to infection. When appropriate, expression plasmids were transfected 48 h after transfection of siRNA.

### Infectivity assay

Bacterial infectivity was tested as previously described (Dumoux et al., 2012).

### MT regrowth assay

At an appropriate time points (0 min, 5 min, 20 min), cells were treated with 10 ng ml^−1^ nocodazole (Sigma-Aldrich) for 4 h at 37°C. Steady-state cells were directly fixed and stained. ‘0 min’ samples were fixed and stained directly after incubation at 4°C for 20 min, whereas ‘5 min’ or ‘20 min’ samples were appropriately incubated further at 37°C in nocodazole-free media prior to fixation and immunolabeling.

### Cell labeling

For immunolabeling, cells were fixed with either cold methanol (5 min on ice) or 4% (w/v) paraformardehyde in cytoskeleton buffer with sucrose (CBS) [supplemented with 10 mM 2-(N-morpholino)ethanesulfonic acid (MES), 138 mM KCl, 3 mM MgCl, 2 mM EGTA and 0.32 M sucrose] for 30 min at room temperature (RT), and permeabilized with cold methanol/ethanol or 0.5% (v/v) Triton-X100 in CB. Primary antibodies used were, anti-IPAM (generous gift from Dan Rockey), anti-CEP170 (Invitrogen), anti-α/β-tubulin (Cytoskeleton), anti-β-tubulin (Millipore), anti-γ-tubulin (Abcam) and anti-pericentrin (Covance), diluted in PBS containing 1% (w/v) bovine serum albumin (BSA) and incubated (3 h, RT) prior to incubation with appropriate secondary antibodies (Invitrogen) in the same buffer (90 min, RT). Samples were mounted using Mowiol. 200 nM rhodamine-coupled Phalloidin (Invitrogen) was added to the primary antibody mixture. Labeling using wheat germ agglutinin conjugated to Alexa-Fluor-594 (45 min, RT) was performed prior to cell permeabilisation. For simultaneous labeling of IPAM, β-tubulin and CEP170, primary antibodies were appropriately diluted in PBS containing 10% (w/v) BSA and secondary antibodies coupled to Alexa-Fluor-488, -546 or -633 selective for mouse IgG subclasses were used. Controls to exclude crossreactivity were performed prior to the experiments.

### Microscopy

Samples were observed using a confocal microscope (TCS Sp5 or Sp8 AOBS; Leica) with an oil-immersion objective (63×, 1.4 NA; Leica) operated in sequential mode. By using the AOBS^®^, emission windows were selected to ensure no leak between channels using equivalent samples labeled with a single fluorophore. Laser reflections were avoided by allowing a distance of at least 10 nm between the laser used for excitation and the start of the corresponding emission window. Optical *z*-sections were collected every 330 nm.

An OMX V3 (API, division of GE Healthcare) was used for super-resolution microscopy. For this technique, coverslips (Zeiss) were mounted using Vectashield instead of Mowiol, and viewed using an oil-immersion objective (100×, 1.4 NA; Olympus). Prior to acquisition, the optical transfer function for each channel was calculated using calibration beads, the color channels aligned, and CCD camera noise corrected. Image acquisition and reconstruction was performed using DeltaVision software.

### Image analysis, 3D reconstruction and quantification

All images were analyzed using Fiji (Eliceiri et al., 2012). Greyscale modifications were applied across all conditions, and thresholds remained constant. Maximum intensity projections (max-projection) of several *z*-sections are required to observe the entire MTOC.

The 3D viewer plugin (Schmid et al., 2010) was used for 3D-reconstruction, and the volume displayed as a surface using a constant threshold. Prior to 3D-reconstruction, images were treated using the rolling-ball-background algorithm plugin with a mean filter of 1.5 pixels. To determine the level of expression, puncta of GFP-IPAM^90-268^ were scored from a maximum projection of the entire volume of the cell. Cells with ten or fewer puncta were considered as cells expressing low levels of GFP-IPAM^90-268^. For cells expressing GFP, cells with a mean pixel intensity <33% of the maximum grey value were considered as low-expressing cells.

For quantification, ∼150 infected cells and 250 uninfected cells per condition were analyzed. Representative images of the different classifications used are shown in supplementary material Fig. S2. Student's *t*-test was applied for statistical analyses.

#### Centrosome positioning and IPAM patches

A mask of the inclusion was generated as described (Dumoux et al., 2012) without the ‘fill hole’ option. The inclusion periphery was detected using the ‘find edges’ option (yellow line) and the outline superimposed back onto the RGB images. Centrosomes (γ-tubulin) in contact with the inclusion periphery were scored manually (supplementary material Fig. S2A).

#### Centrosomal markers

Cells (15±7) within fields of view were imaged and max-projection applied to the entire *z*-stack. After manually tracing cell edges, the number, position and appearance of centrosomal labeling were categorized according to the classification presented in supplementary material Fig. S2B.

#### MT regrowth, low magnification

Cells (15±7) within fields of view were imaged and max-projection applied to the entire *z*-stack. After manually tracing cell edges, the number of MT regrowth foci were counted according to the criteria presented in supplementary material Fig. S2C.

#### MT regrowth, high magnification

In cases where >97% of the cells exhibited mono-foci according to the criteria presented in supplementary material Fig. S2C, cells within fields of view (9±3.5) were imaged at higher magnification, and max-projection applied to the entire *z*-stack. After manually tracing cell edges, MT regrowth foci were assessed manually according to the criteria presented in supplementary material Fig. S2D.

#### Cell shape

Cells (15±7) within fields of view were imaged and max-projection applied to the entire *z*-stack. Confluent cells were excluded. After manually tracing cell edges, the numbers of round or elongated cells were counted according to the criteria presented in supplementary material Fig. S2E.

#### Inclusion shape

DNA was stained and cells (9±3.5) within fields of view imaged. Inverted LUT and grey scale modifications were performed uniformly. Manual quantification was performed according to the criteria presented in supplementary material Fig. S2F.

### Plasmids

pET22b-IPAM was generated by polymerase chain reaction (PCR) amplification of the gene encoding IPAM (*CT223*) from *Chlamydia trachomatis* serovar L2 genomic DNA. The PCR product, engineered to contain an *Nde*I restriction site at the 5′-end of the start codon and a *Xho*I restriction site at the 3′-end of the gene, was cloned into the corresponding sites of the T7 expression vector pET22b (Novagen) creating an in-frame C-terminal fusion of six histidine residues (expected molecular mass: 32 kDa). pEGFP-IPAM^90-268^ encoding the cytoplasmic domain of IPAM (IPAM^90-268^) was generated by PCR amplification of the appropriate fragment of the IPAM (*CT223*) gene from *Chlamydia trachomatis* serovar L2 genomic DNA. The PCR product was engineered to generate an *Eco*RI restriction site followed by a start codon prior to the codon encoding residue 90 of IPAM and a *Kpn*I restriction site at the 3′-end of the stop codon. This was cloned into the corresponding sites of the expression vector pEGFP-C2 (Clonetech) creating an in-frame N-terminal fusion of green fluorescent protein (GFP) (expected molecular mass: 52 kDa).

### Expression and purification of IPAM

pET22b-IPAM *E.coli* C41(DE3) (Miroux and Walker, 1996) transformants were cultured (200 rpm, 37°C) in Luria's broth (LB) containing ampicillin (50 μg ml^−1^) until A_600_∼0.8. Protein expression was induced by using 0.05 mM isopropyl β-D-thiogalactopyranoside (IPTG, Sigma-Aldrich) and incubation continued (2 h, 37°C). Cells were harvested (centrifugation at 2000 ***g***, 15 min, 4°C) and resuspended in Tris-buffered saline (20 mM Tris HCl pH 7.4, 140 mM NaCl; TBS) containing protease inhibitors (Roche), prior to lysis with a pressure cell homogenizer (18,000 psi, Stansted). After clarification (centrifugation at 2000 ***g***, 10 min, 4°C), membranes were separated by centrifugation (31,000 ***g***, 1 h, 4°C). Membranes containing IPAM were incubated with appropriate buffers (TBS, or 20 mM Tris HCl pH 7.4, 1 M NaCl; TBS, 100 mM Na_2_CO_3_ pH 11; 8 M urea) or with 1% (w/v) detergent [ASB-14 (Millipore), Triton X-100 (Sigma-Aldrich), CHAPS (Sigma-Aldrich), dodecyl-β-d-maltoside (Sigma-Aldrich), polyoxyethylene (Lane et al., 2008) tridecyl ether (Sigma-Aldrich), C7BZ0 (Sigma-Aldrich), octyl-β-d-glucopyrone (Sigma-Aldrich) or octyl-β-d-1-thioglucopyrone (Sigma-Aldrich)] in TBS containing protease inhibitors (Roche). Solubilized material was recovered by centrifugation (31,000 ***g***, 1 h, 4°C). Buffer containing 0.7% (w/v) ASB-14 extracted IPAM from the membrane most effectively, and was retained during binding; then, elution from nickel nitrilotriacetic acid-agarose was carried out under native conditions according to the manufacturer's instructions (Qiagen).

### Pull-down assay

Purified IPAM was incubated (10 min, RT) with magnetic beads coupled to cobalt (Dynabeads, Invitrogen) before incubation (45 min, rotating, 30°C) with HeLa cell lysate (10^6^ cells sonicated 20 pulses, 5 s, 18 V, 4°C; Misonix XL-1000 ultrasonicator) in phosphate buffer saline (PBS) containing 175 µg ml^−1^ phenylmethylsulfonide fluoride (PMSF), 5 µg ml^−1^ lysosyme and one mini-complete protease inhibitor tablet. Beads were then washed and stored at −80°C.

### Mass spectrometry

Bead-coupled proteins were digested overnight at 37°C with sequencing grade trypsin (12.5 μg ml^−1^; Promega) in 20 μl 25 mM NH_4_HCO_3_. Digests were analyzed using a LTQ Velos Orbitrap (Thermo Fisher Scientific) coupled to a nano-LC Ultimate 3000 RSLCnano system (Thermo Fisher Scientific). Peptides were analyzed in the orbitrap in full ion scan mode at a resolution of 30,000 (at *m*/*z* 400) and with a mass range of *m*/*z* 400−1800. MS/MS data were searched against the NCBInr *Homo sapiens* and the SwissProt *Chlamydia trachomatis* databases. False discovery rate (FDR) was calculated using the reversed database approach with a 1% filter for strict FDR and 5% for relaxed FDR. The presence of IPAM was verified. The Mascott score was 2395.22, with ten peptides read 2-20 times. A differential analysis was applied to identify targets, using GFP and additional Inc proteins as a reference; a threshold of >30 was applied to the Mascott scores.

### Coimmunoprecipitation

24 h post-transfection, protein-G-coupled magnetic beads (Dynabeads, Invitrogen) were coupled with anti-CEP170 (Invitrogen) or anti-GFP (Invitrogen) antibodies prior to incubation (45 min, rotating, 30°C) with lysate from HeLa cells expressing GFP-IPAM^90-268^ or GFP. After washing with PBS, proteins were eluted using 75 mM Tris HCl pH 7.4, 0.5 M EDTA, 0.05% SDS and mixed with the same volume of loading buffer [80 mM Tris HCl pH 6.8, 20% (v/v) glycerol, 0.16% (w/v) SDS, 20% (v/v) β-mercaptoethanol, 3 mg ml^−1^ bromophenol blue].

### Protein electrophoresis and western blotting

Proteins were analysed by 14% (v/v) SDS-PAGE (Prosieve, Lonza). For gel staining, Coomassie Blue R250 staining was performed. For western blotting, proteins were transferred overnight at 4°C. Nitrocellulose membranes were incubated in TBS, 0.05% (v/v) Tween 20, 5% (w/v) skimmed milk (TBST-milk) for 1 h before an overnight incubation at 4°C with primary antibody diluted in the same buffer. Primary antibodies were anti-GFP (Clontech), anti-CEP170 (Invitrogen), anti-His (Invitrogen) or anti-γ-tubulin (Sigma-Aldrich). Membranes were washed with TBST, incubated for 1 h at RT with appropriate secondary antibodies coupled to horseradish peroxidase (HRP, Invitrogen) in TBST-milk and finally wash in TBST. Chemiluminescence-based immunodetection of HRP was performed according to the manufacturer's instructions (ECL, GE Healthcare). Films (GE Healthcare) were exposed, developed (Xograph) and scanned (Epson).

For densitometry, the protein of interest (CEP170) and a loading control (γ-tubulin) were detected from the same membrane. The ‘gel analysis' algorithm in Fiji (Eliceiri et al., 2012) was used. A double ratio was used to simultaneously correct for loading and to compare with the control:

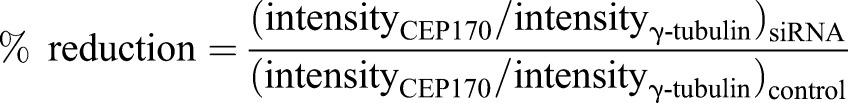


## Supplementary Material

Supplementary information
