# Supplementary material for: A Chlamydia effector recruits CEP170 to reprogram host microtubule organization
Source: J Cell Sci. 2015 Sep 15;128(18):3420–34. doi: 10.1242/jcs.169318 (PMC4582400; doi:10.1242/jcs.169318)
Supplement: Supplementary information [file supp_128_18_3420__index.html]

Supplementary information 

# A *Chlamydia* effector recruits CEP170 to reprogram host microtubule organization

## JCS169318 Supplementary information

**Files in this Data Supplement:**

- Supplementary information
