# Supplementary material for: A Chlamydia effector recruits CEP170 to reprogram host microtubule organization
Source: J Cell Sci. 2015 Sep 15;128(18):3420–34. doi: 10.1242/jcs.169318 (PMC4582400; doi:10.1242/jcs.169318)
Supplement: Supplementary information [file supp_jcs.169318_JCS169318supp.pdf]

Supplementary Figures

	% identical aa	% similar aa
CLIP1	30.9	33.7
pericentrin	24.7	41.6
CEP72	21.3	27.5
TACC1	18.5	15.2
TTDN1	6.7	15

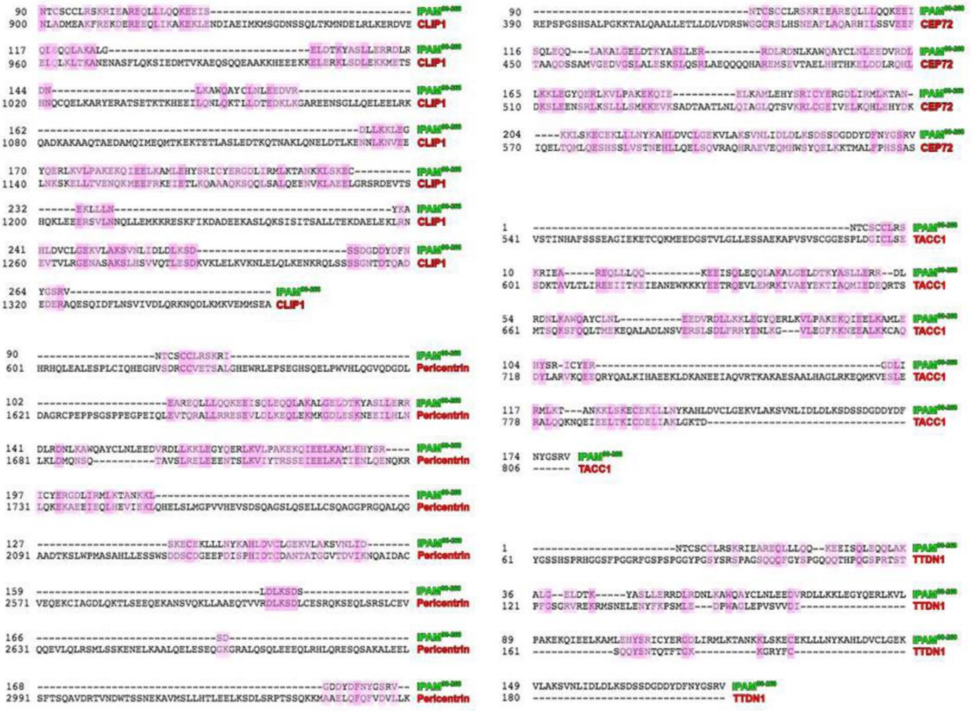

Fig S1: Primary sequence similarity between IPAM$^{90\text{-}268}$ and human centrosomal and microtubule-related proteins.

Table shows amino acid identity and similarity of the top 5 *Homo sapiens* proteins. Panels show pairwise alignments of these 5 proteins with IPAM$^{90\text{-}268}$, where identical amino acids are highlighted in dark purple and similar amino acids in light purple. CLIP1: cytoplasmic linker protein 1, CEP72: centrosomal protein 72kDa, TACC1: transforming-acidic coiled-coil protein, TTD1: trichothiodystrophy protein 1 non-photosensitive.

Journal of Cell Science • Supplementary information

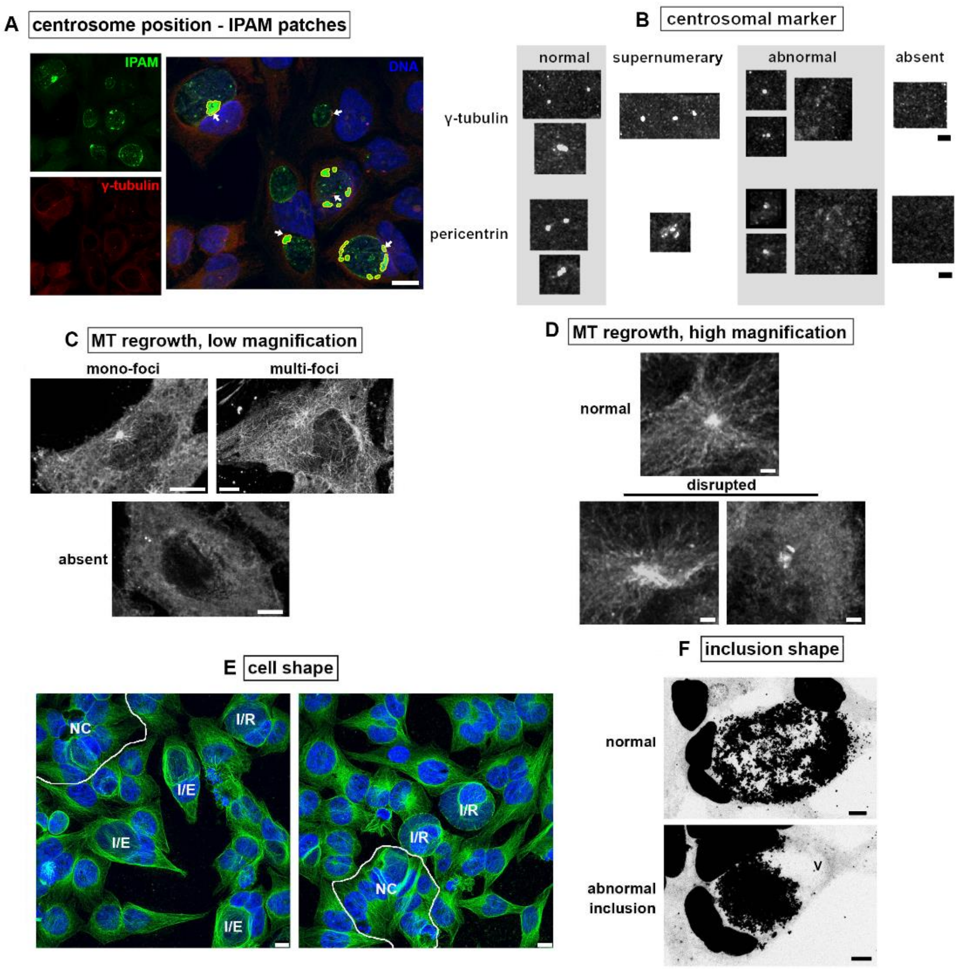

Fig S2: Representative images depicting classes used for quantification.

HeLa cells were treated/infected/subject to MT regrowth assay as appropriate. Cells were labeled for the structure of interest, and confocal z-stacks acquired prior to Max-projection analysis of the whole cell volume. A minimum of 150 infected cells and 250 non-infected cells were quantified per experimental condition. Dividing cells were excluded. Figures show representative images depicting the classes used (**A**) to determine IPAM patches and the positioning of γ-tubulin (**B**) to describe the labeling of γ-tubulin and pericentrin. Scale bar: 2 μm (**C**) to determine the number of foci of MT nucleation. Scale bar: 10μm. (**D**) to determine the features of individual nucleation foci. Scale bar: 5 μm (**E**) to determine cell shape. Cells were labeled to visualize DNA (blue) and microtubules (green). NC: non-considered area due to high cell confluency, I/E: infected elongated cells, I/R: infected round cells. Scale bar: 10μm. (**F**) to assess inclusion organization. Look up table for the nucleic acid channel was inverted. V: void. Scale bar: 10 μm.

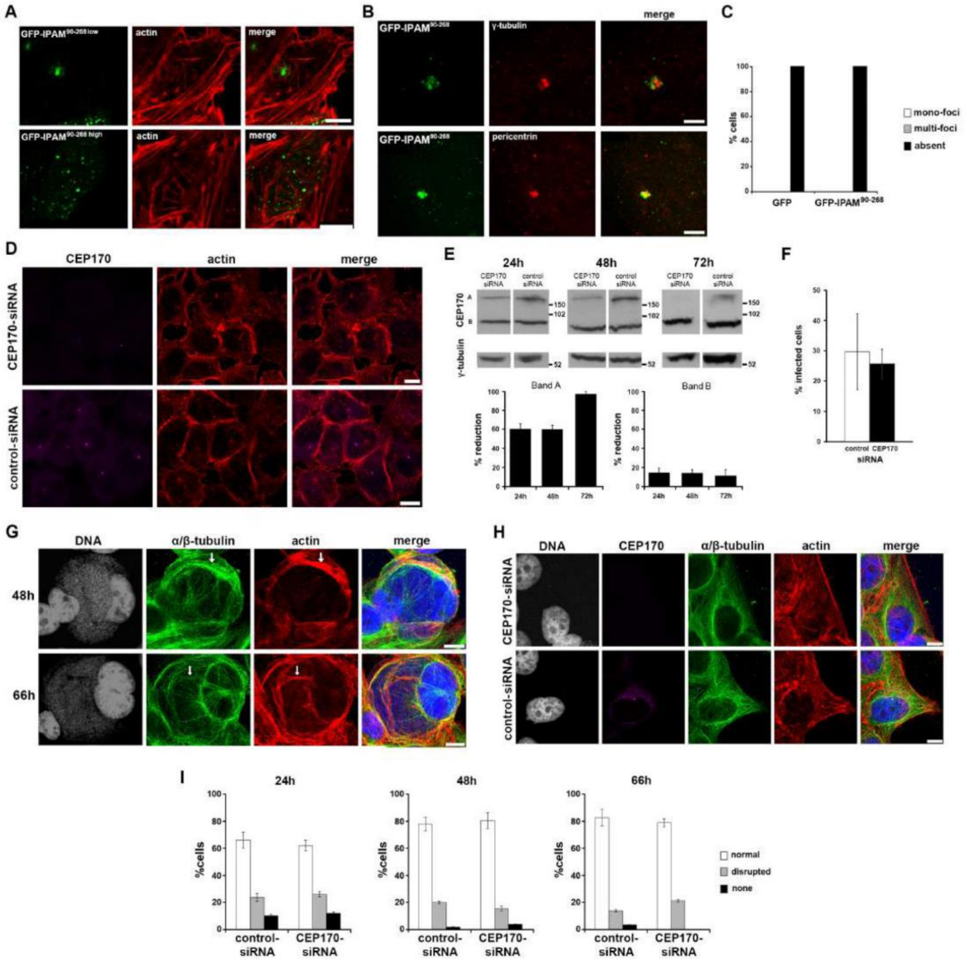

Fig S3: Impact of expression of GFP-IPAM$^{90-268}$ and CEP170 knockdown on cytoskeleton.

(**A**) HeLa cells expressing low (GFP-IPAM$^{90-268\ low}$; top) or high (GFP-IPAM$^{90-268\ high}$; bottom) levels of GFP-IPAM$^{90-268}$ (green) were fixed and stained for F-actin (red).

Journal of Cell Science • Supplementary information

Scale bar: 5 μm. (**B**) MTs were depolymerized in HeLa cells expressing GFP-IPAM$^{90\text{-}268}$ (green) prior to fixation and staining for γ-tubulin or pericentrin (red). Panels show maximum projections of 3 confocal z-sections. Scale bar: 5 μm. (**C**) MTs were depolymerized in HeLa cells expressing GFP or GFP-IPAM$^{90\text{-}268}$, and MT architecture in individual cells categorized (as described in **Fig S2C**). (**D**) HeLa cells were treated with CEP170-siRNA or control siRNA, fixed and stained for CEP170 (magenta) and F-actin (red). Panels show maximum projections of 3 z-sections. Scale bar: 10 μm. (**E**) Immunoblots using anti-CEP170 and anti-γ-tubulin antibodies of HeLa cell lysates following treatment with CEP170-siRNA or control-siRNA for 24-72 h. Histograms show reduction in CEP170 species (bands A and B) corrected for relative loading using γ-tubulin. (**F**) HeLa cells were treated with control-siRNA or CEP170-siRNA and infected with *C.trachomatis* L2. Infectivity was assessed 24 h later. (**G**) HeLa cells were treated with CEP170-siRNA and infected with *C.trachomatis* L2. Cells were fixed 48 h or 66 h later and stained for DNA (grey/blue), α/β-tubulin (green) and actin (red). Panels show maximum projections obtained from the 4 z-sections. Arrows indicate an extensive contact between actin and microtubules. Scale bar: 10μm. (**H**) Non-infected HeLa cells were treated with CEP170-siRNA or control-siRNA, fixed, and co-stained for DNA (grey/blue), CEP170 (magenta) α/β-tubulin (green) and actin (red). Scale bar: 10 μm. (**I**) HeLa cells were treated with CEP170-siRNA or control-siRNA for 24, 48 and 66 h. MTs were depolymerized then allowed to regrow for 5 min prior to fixation and staining for CEP170 and α/β-tubulin. Following confocal imaging, the regrowing MT network in individual cells was categorized (as described in **Fig S2D**). The differences were not statistically significant.

Journal of Cell Science • Supplementary information

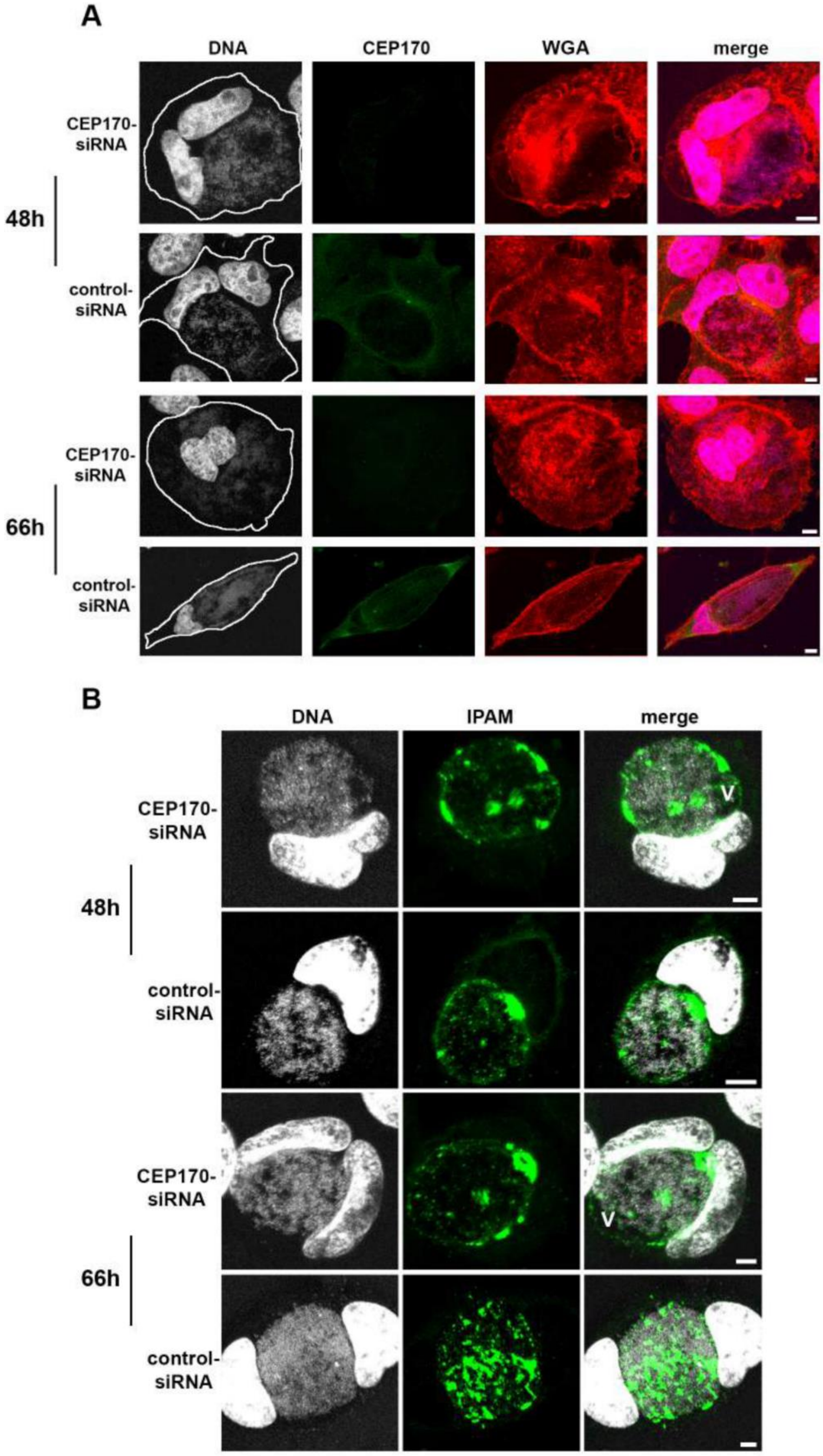

Fig S4: CEP170 is necessary for cell and inclusion shape in cell infected with *C.trachomatis*.

HeLa cells were treated with CEP170-siRNA or control-siRNA and infected with *C.trachomatis* for 48 h or 66h prior to fixation and labeling for (**A**) DNA (grey/magenta), CEP170 (green) and WGA (red) with the cell periphery outlined in white, or (**B**) DNA (grey) and endogenous IPAM (green), v: void. Scale bar: 10 μm.

Journal of Cell Science • Supplementary information
